# Optimizing ensemble machine learning models for accurate liver disease prediction in healthcare

**DOI:** 10.1371/journal.pone.0330899

**Published:** 2025-08-28

**Authors:** W. El Atifi, O. El Rhazouani, Fida Muhammad Khan, H. Sekkat

**Affiliations:** 1 Hassan First University of Settat, High Institute of Health Sciences, Laboratory of Sciences and Health Technologies, Settat, Morocco; 2 Department of Radiotherapy, Hospital Center Ibn Rochd, Faculty of Medicine and Pharmacy, University Hassan II, Casablanca, Morocco; 3 Department of Computer Science, Qurtuba University of Science and Information Technology, Peshawar, Pakistan; Sunway University, MALAYSIA

## Abstract

Liver disease encompasses a range of conditions affecting the liver, including hepatitis, cirrhosis, fatty liver, and liver cancer. It can be caused by infections, alcohol abuse, obesity, or genetic factors, and it often progresses silently until advanced stages. Early detection and lifestyle adjustments are essential for effective management and to prevent severe liver damage. This study explores the application of machine learning (ML) techniques to predict liver disease, leveraging a dataset to compare the performance of several ensemble classifiers. The algorithms include the Random Forrest Classifier, Ada Boost Classifier, and Gradient Boosting Classifier. After a series of feature extraction and selection, hyperparameter tuning by Randomized Search CV and GridSearchCV, we aimed to determine the best model for liver disease prediction in terms of accuracy, precision, recall, and F1-score. The results showed that the Random Forest Classifier, optimized with GridSearchCV, achieved the highest accuracy at just over 85.17%. The considerations presented in this classifier can be considered for potential use as a precise diagnostic tool for liver disease diagnostics as these measurements indicate that this classifier works balanced with precision at 0.85 for both the presence and absence of the given disease as well as recall of 0.81 for its presence and 0.87 for its absence and F1-measure of 0.83 and 0.85 respectively. There were also relatively high performances of AdaBoost Classifier and Gradient Boosting Classifier, though none of the classifiers outperformed Random Forest Classifier significantly. The research has shown the potential of ensemble ML techniques, especially in the diagnosis of medical conditions, including liver diseases which, if diagnosed early, are critical. The results add evidence regarding the applicability of the ML models in clinical practices with the potential to improve diagnostic activities and consequently the outcomes of patients. Future studies will build on these models, testing them on larger and more diverse sets of data, including aspects of deep learning, and apply the research to other disease domains. The work presented in this research offers a starting point for carrying out innovations with ML in the sphere of healthcare to progress the methods of diagnosing diseases and treatment.

## 1. Introduction

Chronic liver disease, in particular, is a major source of disease and mortality globally and affects a wide range of populations. It covers a whole range of diseases such as viral hepatitis, alcoholic liver diseases, non-alcoholic fatty liver diseases (NAFLD), cirrhosis, and hepatocellular carcinoma (HCC). Getting an accurate diagnosis of liver diseases is very important for patients because early diagnosis and treatment can help prevent further damage. Nevertheless, most such problems with the liver are not easily diagnosable due to their complications and the limitations of general diagnostic tools. In recent years, big data has served as a beneficial factor for the prediction and classification of many diseases, such as liver diseases, by using ML techniques. By so doing, most advanced computational concepts in what is popularly referred to as MH or Medical Hematology can use large volumes of data, especially clinical and laboratory data, to find patterns and relationships that may not be evident to even the most experienced clinicians. This makes it easier for physicians, nurses, and all healthcare professionals to get more insights into how they diagnose diseases and treat them. Currently, the trend in the use of machine learning in hepatology is steadily growing, and due to this fact, the accuracy of diagnosing liver pathology, risk assessment, and the choice of therapy tactics can be improved. The authors discussed the importance of machine learning in predicting and classifying liver diseases and its importance in pushing hepatology forward. In this section, we will highlight reasons why the diagnosis of liver diseases remains complex and why it is beneficial to rely on the application of ML approaches and techniques in this area, together with the methods used in this regard.

1. **Challenges in Liver Disease Diagnosis:** Liver diseases remain one of the most difficult to diagnose effectively because of the many varieties of causative agents, and because the disease is frequently asymptomatic in the early stages. Serum biochemical tests that include liver function tests, ultrasonography, CT scan, MRI, and liver biopsy also have severe limitations in terms of sensitivity and specificity in diagnosing NAFLD. They can lead to delays in the assessment or, on occasion, incorrect diagnoses that could conceivably hinder one’s timely management and containment of diseases. Therefore, the demand for enhanced techniques that can better identify human disease is increasing.
2. **Advantages of Machine Learning in Liver Disease Diagnosis:** Several advantages are given to machine learning in the context of diagnosing liver disease. State-of-the-art ML algorithms can process large datasets from clinical, laboratory, and imaging domains. They do a remarkable job of detecting non-linear associations and structures within this data, by which conventional statistical methods often fail. However, as new knowledge and standards in medicine are developed, the specifics of the used algorithms can be changed to correspond to new knowledge and used by models to make diagnoses.
3. **Methodologies and Approaches:** In liver disease prediction and classification, a typical workflow involves data collection, preprocessing, feature selection, model training, evaluation, and deployment. Various machine learning algorithms can be employed, including logistic regression, decision trees, random forests, support vector machines (SVM), and gradient boosting methods like XGBoost. The choice of the most suitable algorithm depends on the specific characteristics of the dataset and the research objectives.

The liver is one of the most critical organs of the human body. It plays an essential role in the body’s function. Primary purposes include removing toxins from the body, fighting against infections, and balancing the hormones and secretion of bile juice [1]. If these functions are not performed by the liver correctly, it will result in several complications and liver diseases. Therefore, if a virus infects the liver, chemicals that injure the liver are consumed, or the immune system’s dysfunction occurs, severe damage to the liver or malfunctioning may happen, which ultimately might cause death [2]. Liver disease is one of the most chronic and threatening diseases globally that can cause various side effects if not treated early [3]. According to the World Health Organization (WHO) report in 2018, the number of deaths due to liver diseases is around one million, and ranked 11th in the world with a critical number of fatalities. As the symptoms of liver disease cannot be visible until the condition becomes chronic, it is challenging and daunting for healthcare professionals to identify liver disease at its early stages [4]. In addition, the traditional testing methods like sonography, MRI scans, and CT scans that are available for detecting liver diseases are expensive and harmful, with numerous side effects [5].

Thus, a significant constraint found by healthcare workers is to predict liver diseases at an early stage, at minimal cost, and at the same time provide a better healthcare system to treat liver diseases. Severe liver diseases include problems with indigestion, dry mouth, pain in the abdomen, skin color turning yellow, numbness, memory loss, and fainting [6]. Unnoticed at the initial stages, these symptoms are only visible when the disease turns chronic. However, even though the liver is partially infected, it can still function [7]. Diagnosis of liver diseases can be divided into three stages, i.e., the first stage is liver inflammation, the second is liver scarring (cirrhosis), and the final stage is liver cancer or failure. Since these scenarios are present in liver disease, early prediction is significant in providing better health for New Zealanders. If liver disease is diagnosed early, there will be a chance of early treatment and control of deaths due to liver disease [8]. But when the liver fails to function, few treatments are available except liver transplantation [9], which is very expensive, particularly in New Zealand (Hepatitis C, 2021). Apparently, in New Zealand, 35–40% of the population is not diagnosed with Hepatitis C at the early stages because of the asymptomatic behavior of liver disease. Unfortunately, most of these individuals do not know the risks linked to liver disease. Due to the asymptomatic behavior and higher costs of liver disease treatment, it is essential to prevent or diagnose liver disease early for better treatment. With advancements in biomedical sciences, the healthcare system has significantly improved by predicting disease using machine learning techniques [10]. Machine Learning algorithms are one of the potential solutions to this problem due to their handling of large amounts of data and employing different approaches like classification, association, and clustering, which benefits in realistic arbitration of disease prediction [11]. There are different learning techniques in ML methods, one of which is supervised learning. Supervised learning techniques use labeled data and map the input and output data. These supervised learning methods are widely used for prediction and classification [12]. Supervised learning techniques would be appropriate as this research predicts whether the patient has liver disease or does not have liver disease. With increasing interest in using machine learning approaches in diagnosing liver diseases, various research papers have been published showing the effectiveness of these methods. Some of these works include [13], where random forests were used to accurately predict cirrhosis in NAFLD patients [14] where Deep learning models were applied for liver disease-related pathologies in medical image analysis.

This research study was followed by a detailed investigation of machine learning techniques in the prediction and classification of liver diseases. This paper’s remaining sections review details of data collection and preprocessing, model selection and evaluation, some key issues on the ethical use of these tools, and the significance of the synergy between data scientists and medical experts in realizing the potential of these innovative tools for enhanced patient care in hepatology. In this regard, early diagnosis of liver diseases remains a significant challenge due to constraints in the effectiveness of the conventional diagnostic approach. Promising results demonstrate that machine learning will benefit liver disease diagnosis, yielding new challenges including data collection of high-quality data sets, decision on the correct algorithm for diagnosis, interpretability of the diagnosis results, and ethical issues. The challenge is to derive machine learning models that can overcome the said challenges and deliver credible and ethical diagnostic aids for clinicians in valid practical environments.

*The research contribution of this research study is given below:*

- To evaluate the performance of various ensemble machine learning classifiers (Random Forest, AdaBoost, and Gradient Boosting) in predicting liver disease, focusing on metrics such as accuracy, precision, recall, and F1-score, to determine the most effective model for early detection of liver conditions.
- To optimize the selected machine learning models using feature selection and extraction, selection techniques, and hyperparameter tuning through RandomizedSearchCV and GridSearchCV, with the aim of enhancing predictive accuracy and balancing performance metrics for liver disease diagnosis.
- To assess the applicability of machine learning models in clinical practice for liver disease diagnosis, exploring their potential to improve diagnostic outcomes and contribute to better patient management, while identifying future research directions for integrating deep learning techniques and expanding to other medical domains.

## 2. Literature review

Liver diseases are a category of diseases that affect the liver, such as viral hepatitis, alcoholic diseases, non-alcoholic fatty liver diseases (NAFLD), cirrhosis, and hepatocellular carcinoma (HCC). Any impairment in the liver is a major health issue because the liver is responsible for the metabolism of nutrients, detoxification of blood, synthesis of proteins, and control of coagulation factors Altaf et al., 2022) [15]. The global prevalence of liver disease is high, and the cause could be attributed to alcoholism, obesity, and viral hepatitis. According to the World Health Organization (WHO) global hepatology statistics, liver disease-related deaths are common across the globe; they comprise cirrhosis and liver cancer. Currently, there are several diagnostic approaches to liver diseases; they include clinical chemistry (liver function tests), imaging (ultrasound and CT scan, MRI, and fibroscopy (liver biopsy). Although these methods are useful, their weak points are their sensitivity, specificity, and capacity to detect early-stage diseases Md et al., 2023) [16].

New advancements in ML provide possible solutions to enhance the diagnosis and prognosis of liver disorders. By bringing in powerful data analysis tools, an ML algorithm can work through datasets, allowing for the identification of determinable patterns and relationships that may be overlooked by clinicians. Viral Hepatitis: There has been the use of ML in chronic diseases such as hepatitis C, where researchers utilize clinical and demographic information to determine patients apt to benefit from antiviral therapy Aberg et al., 2023) [17]. Some applications of ML models include NAFL and NASH, and creating a machine learning system to diagnose the severity of NASH without using biopsies Khan et al., 2022) [18]. An earlier study reported that the use of the ML approach in detecting early-stage HCC using anatomical and functional imaging data is promising, and some of these models have higher accuracy in differentiating between HCC and benign liver lesions Niu et al., 2022) [19].

The diagnosis and treatment of liver diseases remain one of the major challenges confronting hepatologists due to the complexity of disorders observed in the liver and due to current-day diagnostic capabilities. Conventional diagnostic tools for the assessment of hepatic diseases include biochemistry, LFTs, and imaging modalities such as ultrasound, CT, and MRILiver biopsies. But such methods are not without certain drawbacks. For example, liver function tests that are essential during the primary assessment can give an inaccurate presentation of the severity of liver injury or early disease due to their inter-assay variability and non-site specificity Gao et al., 2023) [20], REDDY et al. (2024) [21]. Imaging techniques, though helpful with anatomical information, fail to delineate the early fibrotic stage from other stages of liver disease or when they are applied to determine the nature of the lesion without a biopsy Ali et al., 2024) [22]. Liver biopsy, which is the gold standard for staging liver fibrosis and cirrhosis, is invasive, poses possible risks, and is prone to sampling error Trebicka et al., 2022) [23].

New horizons have therefore opened up in the field of liver disease diagnosis and prognosis by incorporating ML techniques in medical diagnosis. Traditional diagnosing methods, however, have their inherent flaws; yet, there are ML algorithms that, with the help of big data and pattern-recognizing capabilities, may provide a solution. For example, in viral hepatitis, there are ML models like support vector machines that have been applied to predict treatment response, which may serve as an invasive and more efficient method of making treatment decisions than invasive procedures Prakash et al., 2023) [24]. Likewise, in NAFLD/NASH, the ML models have put a possibility in differentiating the two diseases, which may help in the future to decrease the utilization of liver biopsies for staging the diseases Xu et al., 2022) [25].

Furthermore, ML has a high capability of identifying hepatocellular carcinoma, which is one of the leading causes of cancer deaths in the world. Analyses based on convolutional neural networks (CNN) display the effectiveness in accurately detecting early-stage HCC in imaging data and show higher sensitivity and specificity than conventional diagnostic methods Doghish et al., 2023) [26]. Such developments highlight the fact that the application of ML can go a long way to revolutionize early diagnosis and classification of liver diseases and therefore offer direction towards a more effective management of patient care.

However, it is still not an easy task to implement ML into the clinical hepatology stream comprehensively. The effectiveness of ML models is very much dependent on the quality and variety of the dataset used to train the model, and there are problems of data scarcity and representativeness Toyoda et al., 2022) [27]. In addition, there is a major limitation of opacity, of which many ML algorithms are black-boxes, to gain clinical acceptance, there is a need to immerse in building more explainable models Farber et al., 2023) [28]. Other factors that need to be incorporated include the workflow, available regulations, and ethical issues; hence, effective clinical integration when achieved must therefore be the result of a concerted effort between clinicians, data scientists, and policymakers Semmler et al., 2022) [29].

Although the study conducted by Ahn et al. (2021) [30] suggested the use of Liver disease prediction by applying both methods namely SVM and Naïve Bayes utilizing MATLAB 2013 software on a data set known as Indian Liver Patient Records having 583 instances and 11 attributes with the accuracies 79.66% for SVM and 61.28% for Naïve Bayes. In their findings, they reveal that the time taken to execute SVM was 3210ms, two times the time taken by Naïve Bayes (i.e., 1670ms), not pre-processing missing values. Besides the accuracy, they realized that SVM surpassed Naïve Bayes. Lee et al. (2021) [31] also correctly predicted liver disease using various ML techniques such as SVM, Random Forest, Decision Trees, Artificial Intelligence, and Naïve Bayes. The research was run in R using the Indian Liver Patient Records dataset, which has 11 features with 583 instances. These accuracies were attained from Support Vector Machine (SVM) being 77%, Random Forest at 77%, Decision Trees at 81%, Artificial Intelligence at 71%, and Naïve Bayes at 37% respectively, with Decision Trees having the highest accuracy and Naïve Bayes having the least of the set. Ajmera et al. (2022) [32] made a prediction analysis on patients who have Fatty Liver Disease (FLD). The research collected 700 patient records from New Taipei Hospital, which had screening tests for fatty liver disease; out of 700 patients, 577 records were considered, depending on the patient’s age and sufficient data. Of those 577 patients, 377 had fatty liver disease, and the remaining had no fatty liver disease. The dataset contains patient health details of age, gender, systolic and diastolic blood pressure, abdominal girth, glucose level, triglyceride, HDL-C, SGOT-AST, and SGPT-ALT. The Synthetic Minority Over-Sampling Technique (SMOTE) was applied at the data preprocessing stage, and normalization was done. Four ML algorithms, namely Random Forest, Naïve Bayes, Artificial Neural Network, and Logistic Regression with 3, 5, and 10-fold cross-validation, were applied in the next step. In addition to the accuracies, the area under the receiver operating characteristic curve for all the algorithms was observed. Random Forest gave the best accuracy across all the cross-validations from all the results.

Nahar et al. (2023) [33] focused their research on predicting liver disease using different classification methods with feature selection and implementing software for easy prediction. The study was conducted on the Indian Liver Patient Records dataset. Some attributes were removed during the feature selection phase using the Correlation-based Feature Selection Subset Evaluator with the Greedy Stepwise search method in WEKA. Only five (5) attributes were selected through this method: Total Bilirubin, Direct Bilirubin, Alkaline Phosphatase, Alamine Aminotransferase, and Aspartate Aminotransferase. With this, six different classification methods were applied: Logistic Regression, Naïve Bayes, Sequential Minimal Optimization (SMO), Random Forest, Instant based Classification (IBk), and Logistic Regression provided the highest accuracy with 74.36%. The lowest accuracy was produced by Naïve Bayes (55.9%).

In this study, we proposed the optimistic outcomes from machine learning methods applied in the prediction of liver diseases on the Indian liver patient records dataset, as used in various studies. To improve the predictive values of the algorithms and increase the diagnostic sensitivity and specificity of the diagnostic classifiers, we propose the usage of RF, AdaBoost, and Gradient Boosting algorithms in conjunction with Grid SearchCV for hyperparameter tuning for early diagnosis of liver diseases. Via optimizing feature extraction, tuning the hyperparameters, and using larger and more diverse datasets, our work will investigate whether these models could be migrated into the clinic. Furthermore, we will explore concerns about incorporating improved categorization utilizing machine learning methods to extend the viability of the proposed approach to other disease categories and ML-based healthcare applications.

## 3. Research methodology

Our research methodology encompasses a comprehensive approach to developing and evaluating machine learning models for liver disease prediction.

### 3.1 Liver disease dataset collected from kaggle

We extract our data from the Kaggle website, which has a large collection of benchmark data sets and a data science competition platform. The variable contained in this dataset consists of clinical and biochemical parameters of patients such as age, sex, total bilirubin, direct bilirubin, alkaline phosphatase, alanine aminotransferase, aspartate aminotransferase, total proteins, albumin, the albumin/globulin ratio, and a target variable of liver disease as 0 and 1(Table 1). The dataset is considered the most appropriate and the only one that can provide comprehensive information to achieve the study objectives.

**Table 1:** Dataset parameter/attributes.

Attribute	Description
Age	The age of the patient.
Gender	The gender of the patient (Categorical: Male or Female).
Total_Bilirubin	Total bilirubin level in the blood.
Direct_Bilirubin	Direct bilirubin level in the blood.
Alkaline_Phosphatase	Alkaline Phosphatase enzyme levels.
Alamine_Aminotransferase	Alanine Aminotransferase (ALT) enzyme levels.
Aspartate_Aminotransferase	Aspartate Aminotransferase (AST) enzyme levels.
Total_Proteins	Total protein levels in the blood.
Albumin	Albumin levels in the blood.
Albumin_and_Globulin_Ratio	Ratio of Albumin and Globulin levels.
Dataset	A binary indicator of liver disease (1 for Yes, 0 for No).

### 3.2 Proposed architecture.

### 3.3 Data preprocessing

**Fig 1:**
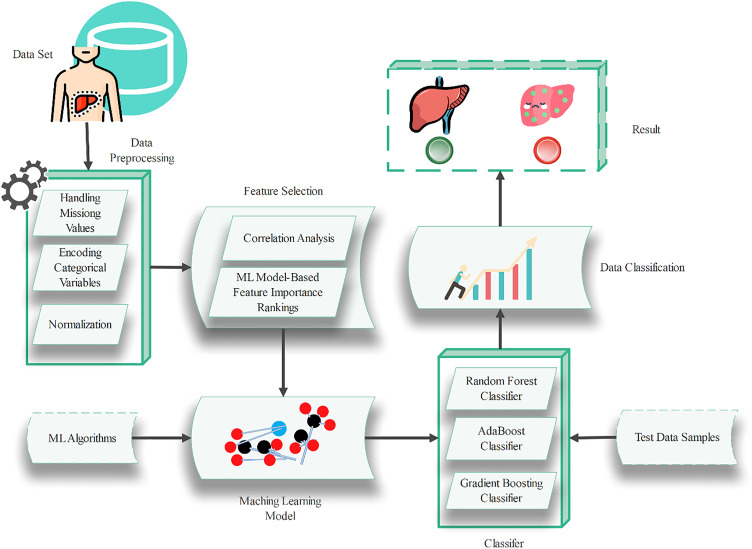
Proposed architecture.

During the preparatory step in data mining, the most important activity is data cleansing and normalization of the dataset for further consumption by the machine learning algorithms (Fig 1). This involves several key steps:

**Handling Missing Values:** All cases of missing values in the current dataset either undergo imputation or are removed from the dataset based on the severity of their value in distorting the data. The record contains many observation variables and special algorithms, for example, mean, median, or mode, are used to complete missing values, if any. On the other hand, if these missing values are considered important or cannot be estimated, then the rows or columns containing these missing values are dropped.

**Encoding Categorical Variables:** Nominal variables, including gender, are transformed into ordinal scales for smooth integration of their data into the ML algorithms. The procedure of transforming categorical features into numerical form is called encoding, and normally, it can be performed, for example, by using one-hot encoding or label encoding. For example, in the case of the categorical variable, such as gender, where the results have to be male/female, they get coded as 0/1 to be solved arithmetically.

**Normalization:** Scaling is used to bring all the features into one scale since most of the features are in different scales. It gives the features proportional importance to the analysis rather than having different magnitudes that may cause some features to dominate others. The number of normalization techniques is fairly limited, but two well-known techniques are min-max Normalization and z-score standardization; the former scales all feature values to a single specified range, and the latter transforms feature values in a way that they have a mean of 0 and a standard deviation of 1.

In performing all the preprocessing steps mentioned above, firstly, it maintains the quality and quality of the data; secondly, it organizes categorical data in a way that makes it easier for machine learning algorithms to compare them; and lastly, it scales the numerical variables so that the learning algorithms give the best results. This helps in arranging the data to be used in subsequent training and testing of various models to empower predictions and determination of liver disease diagnosis and its categorization.

### 3.4 Feature selection and extraction

We proceed to feature selection in our proposed methodology. The goal is to identify the most discriminative features that have a greater impact on predicting liver disease. This process is critical in attaining the best outcome prediction model by removing unimportant, less relevant, or noisy features from the model, making it more simplistic, understandable, and hence interpretable. We describe the feature selection process employed to identify the most important features for predicting liver disease. First, we conducted a correlation analysis to calculate the Pearson correlation coefficient between each feature and the target variable (liver disease status). This analysis helped us identify features that were strongly associated with the target variable. In addition, we used machine learning model-based feature importance, leveraging classifiers such as Random Forest, AdaBoost, and Gradient Boosting. These models provided rankings of feature importance based on their contribution to accurate predictions. The following features were identified as the most important for predicting liver disease: Total Bilirubin, Direct Bilirubin, Alkaline Phosphatase, Alanine Aminotransferase (ALT), Aspartate Aminotransferase (AST), Albumin, and the Albumin and Globulin Ratio. These features were retained for further model training due to their strong relevance to liver disease prediction. The feature selection strategy incorporates two main approaches:

**Correlation Analysis:** The correlation between each feature and the target variable is calculated. The present features that are most likely to have good associations with the target value as potentially beneficial for forecasting. On the other hand, features with little or no association may be considered to be inconsequential for modeling. Also, it can have the problem of redundant features, in which two or more features are strongly related; one of them can be eliminated to avoid multicolor near, which can affect the performance of a model.

**Machine Learning Model-Based Feature Importance Rankings:** Most of the machine learning models, including this study, Random Forest, AdaBoost, and Gradient Boosting classifiers, offer a ranking of feature importance integral to their training process. These models determine the relative importance of each feature in defining the prediction of a model among the features. The performance of these models includes information about the significance of the features, which helps in retaining the most effective features and neglecting the features with a low impact on the results.

Usually, this dual approach not only helps in increasing the predictive accuracy of the models by emphasizing features that are most important but is also advantageous for computational considerations since it decreases the dimensionality of the dataset. Therefore, the models will have better characteristics, which are faster training time and, in general, high potential to generalize to new data. The selected features, having passed through the process of feature selection to distinguish their ability to predict the different parameters, provide the foundation for the construction of accurate and reliable machine learning algorithms for the prediction of liver disease.

### 3.5 Machine learning classifiers

In this research study, we utilize the strength of the ensemble machine learning classifiers to give a much-needed window into the potential and capability of recognizing liver disease. These classifiers are selected because of their ability to effectively solve problems of classification in diverse domains, including health care. Below is a detailed exploration of each classifier utilized in this research:

**Random forest classifier:** The Random Forest Classifier is also based on the bagging technique, which means bootstrap aggregating of decision trees; the different trees are built with different training subsets obtained from the same initial data set. It minimizes the variance and thus avoids overfitting, a major weakness in decision tree-based approaches. Every tree in the forest provides its forecast, and the results are decided by voting, particularly in classification problems [34]. This classifier is most useful when it comes to large feature spaces, outliers in data, and the ranking of features, which is very effective when it comes to the analysis of the results.

**AdaBoost classifier:** AdaBoost is a boosting procedure, short for adaptive boosting, which aims at converting a number of weak learners into a powerful classifier. Instead, the weighting approach works in cycles, adjusting the weights of the separate instances that were classified incorrectly; in so doing, subsequent classifiers pay more attention to difficult instances. This process goes on until one has developed a fixed number of weak classifiers, or until the perfect classifier model is developed. AdaBoost is considered simple and efficient, and it enhances the accuracy of any learning algorithm that it uses [35]. This is especially useful when working with an open dataset where the distribution of data in two categories is skewed, which is the case with liver disease, where the probability of disease could be low amongst the population.

**Gradient boosting classifier:** Yet another less trivial ensemble technique is Gradient Boosting, where models are learning sequentially and each subsequent model is aimed at reducing residuals of the previous models. As opposed to AdaBoost, which modifies instance weights, Gradient boosting aims at fitting new models to the remaining errors created by the preceding models. It enables no additional assumptions about the data and thus can be used to solve a great variety of problems like regression and classification whilst still applying arbitrary differentiable loss functions therefore has a high degree of flexibility [36]. Gradient Boosting has been applauded for the accuracy of prediction and the ability to cope with heterogeneous features and structures that are inherent in a dataset, therefore perfectly suitable for the prediction of liver disease given clinical and biochemical data.

Through the usage of these complex classifiers, our study seeks to capitalize on the elevated precision attained by each of them to give highly accurate and accurate estimations of liver disease. Every classifier has its pros, while Random Forest is less prone to overfitting, AdaBoost addresses the training instances with the most difficulty, is called adaptive; Gradient Boosting is accurate and flexible. This guarantees computed comprehensive examination capable of handling the complexities of liver disease prediction with higher standards of accuracy and procedural efficiency.

### 3.6 Hyperparameter optimization

This paper focuses on Hyperparameter tuning, which is a crucial part of the machine-learning process that seeks to optimize the performance of models by modifying the parameter settings. For the Random Forest Classifier, an ensemble method known for its robustness and versatility in various classification tasks, we employ two widely recognized techniques for hyperparameter optimization: Two of these methods are named Randomized SearchCV and Grid SearchCV.

**Randomized SearchCV:** Randomized SearchCV is more suitable as it randomly chooses a subset of parameters from a specific hyperparameter space for optimization. It enables a practitioner to scan the entire parameter space, which, therefore, is an appropriate approximate to the exact method in terms of practicality and is advantageous to exhaustive search in terms of computational bounds. Random hyperparameter selection with subsequent tuning of a candidate function permits the definition of good configurations compared to the direct search of every possible combination, thanks to the Randomized SearchCV procedure. This is especially useful when the amount of data is large or computational capabilities are constrained, as this service cuts the amount of time required to search for the correct hyperparameters by at least half while offering a reasonable estimate for the optimal hyperparameters.

**Grid SearchCV:** After finalizing the hyperparameters using Randomized SearchCV we again perform the hyperparameter tuning using Grid SearchCV. Being different from Randomized SearchCV, Grid SearchCV does a full search, certainly, about the required parameters within the given parameter grid and examines every combination of the corresponding hyperparameters specified in the grid. This results in the specification of all the best parameters for the Random Forest model, though at a greater computational expense. Importantly, Grid SearchCV is more useful when the space of parameters to search is comparatively small or when the highest model accuracy is expected in the shortest time possible, without being concerned with time consumption.

The results obtained through the usage of both Randomized SearchCV and Grid SearchCV in a two-step manner are expected to be optimized in terms of both computational cost and the quality of the hyperparameters selected for the Random Forest Classifier. Such an approach allows for a broad scanning of the hyperparameter space with Randomized SearchCV to discard a significant portion of irrelevant hyperparameters in the shortest amount of time, and then do a precise scanning of the neighborhood with Grid SearchCV. This systematic approach the hyperparameter tuning aims to improve and strengthen the forecast ability and robustness of the Random Forest model and to make better and more precise scenarios of the liver disease prediction.

### 3.7 Model training

Actual model training is an important step in the implementation of our approach, where each of the specified machine learning categories of classifiers, namely Random Forest, AdaBoost, and Gradient Boosting, are consciously trained on the preprocessed dataset. This step proves valuable for creating models that could be used to forecast liver disease, relying on many factors that define the patient’s condition, namely clinical and biochemical.

**Training and validation:** Split to commence the training process, we first divide the dataset into two distinct sets: one set is left with the HTTP protocol without any changes, using a training set and a validation set. Usually, the dataset is split in a way that the distribution of data in both sets is almost similar, and this may have a ratio of 80, 20 but this depends on the size of the data and the specific research of the study. Consequently, while training the models, it is possible to assign a certain portion of the available data to be used solely for their training with no interference from the programmer, and the other portion predestined solely for the evaluation of the models’ performance as a means of controlling the extent of overfitting and the generalization of the models.

**Model training process:** During training, each classifier is given the selected features paired with the target variable, in this case, the presence or absence of liver disease. In this stage, the models can learn the various features of the given data as well as the correlations between these features and the target variable. In the case of ensemble methods such as those used in this study, the process of building the final model involves creating a large number of decision trees (in the case of Random Forests and Gradient Boosting) or iteratively adding more weak learners (in the case of AdaBoost), which each adapts the parameters of the model in that stage, to make the model better fit the training data.

**Performance evaluation:** It is in the training phase that the validation set becomes an extremely important part, in that it affords a way of assessing the performance of any model being built. Each of the models then provides the predictions on the validation set, and these are compared with the real outcomes to compute the performance of the models by determining the accuracy measure, precision, recall, and F1 measure. These measures provide information on the predictive performances of these models while at the same time avoiding overfitting, especially since they focus on the generality of the models for unseen data.

**Iterative refinement dependent:** Upon the performance assessments, models may still need extra rounds to be trained and perfected. This may be done by going back to the process of hyperparameter tuning to set new configurations for the models or feature engineering to set new inputs. The goal is to repeatedly update the models until their performance strikes the best complexity that would allow them to predict the unseen data accurately.

## 4. Results and discussion

This section describes the outcome achieved by applying the aforementioned methodological approach to employing machine learning classifiers for the prediction of liver disease.

### 4.1 Import the necessary libraries

In the Python programming language, data analytics and machine learning, several preprocessing libraries play a major role in data handling, analyzing, and visualizing capabilities. Pandas are widely known as being used specifically for data structures that are optimal for data manipulation and exploration, specifically features used for manipulating the datasets in the form of tables and data frames. NumPy is related to pandas in that it is an essential extension of pandas, as it supports computation and has features that allow it to handle complex multidimensional arrays and incorporate numerical calculations. Data visualization with Seaborn is a high-level interface to produce statistically sound and beautiful graphics for EDA. On the other hand, matplotlib.pyplot is a unique assortment of functions for creating static, interactive, and animated plots. Taken together, they make up a solid set of libraries that will help data scientists and analysts to find patterns and insights in data, pre-process data for machine learning models, and explain results using simple and interesting visualizations.

### 4.2 Analyzing the distribution of liver disease in patients

**Fig 2:**
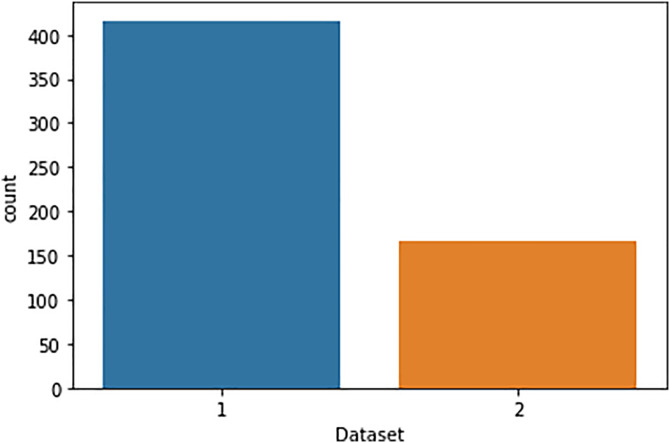
Target variable distribution.

In the exploratory data analysis of our collected dataset, the goal is to identify patient characteristics that can split the patients based on their liver disease status, and that’s why the ‘Dataset’ feature can be considered as the target variable in our research. There are 416 respondents diagnosed with liver disease labeled as ‘1’ and 167 respondents tested negative for liver disease labeled as ‘2’ (Fig 2). This categorization is important for our study, which focused on studying the incidence and distribution of liver disease among the investigated population. Here, we implement data manipulation, as it is crucial for ranking, using the pandas data frame and working with the value_counts() method to count the number of instances within the ‘Dataset’ feature. This allows us to get the right number of patients in each of these groups as a common measure of admission to study them. Besides, to demonstrate how the status of liver disease is distributed among the patients, we employ a Seaborn countplot. This visualization technique generates a bar plot which represents the count of observations in each category, giving the immediate impression of the ratio of occurrence of liver disease and non-liver disease patients in the given dataset. This type of analysis is useful when approaching the exploratory data analysis phase because it provides a visual and quantitative understanding of what the dataset contains or doesn’t and can inform future analytical and predictive modeling processes.

### 4.3 Visualizing age distribution among patients

**Fig 3:**
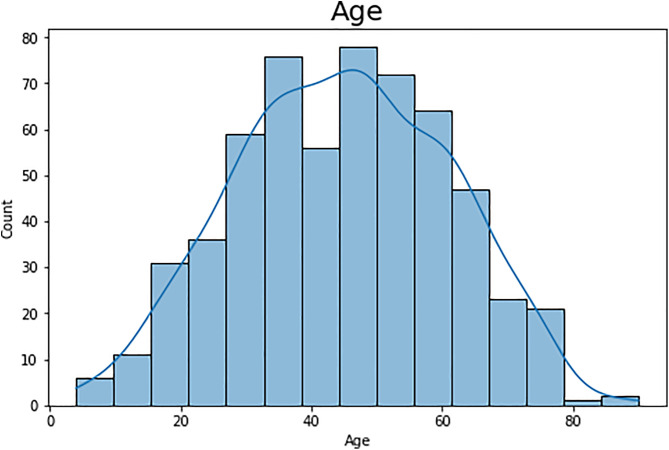
Age distribution among patients.

In this research work, for better visualization and understanding, we plot the histogram of the age of patients in our data set using two tools, matplotlib and Seaborn (Fig 3). To facilitate this observation, we adjust the figure size to be 8 by 5 inches so that the histogram can be observed in detail. As Seaborn’s histplot function, we set the ‘Age’ feature for plotting and enabled the Kernel Density Estimate (KDE) to show the approximation of the age distribution curve. This approach goes further to show the proportions of different ages of patients as well as the distribution of the age by illustrating its nature, for instance, skewed or bell-shaped, or where it is likely to be. The histogram is the primary tool utilized in exploratory data analysis; it is labeled “Age” in a large font to provide information on demographics and to correctly, or at least sufficiently, identify groups at higher risk for liver disease. This visualization is helpful in our research on liver disease because it aids additional analysis and prognoses as well.

### 4.4 Exploring gender disparities in liver disease

**Fig 4:**
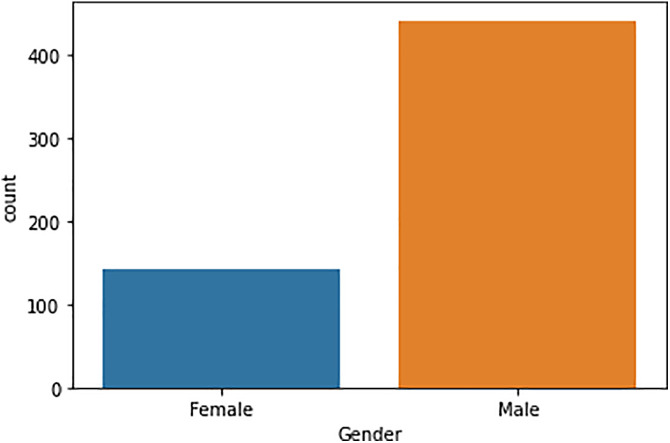
Gender classification.

In our dataset analysis, we meticulously examine the distribution of genders, highlighting the division between male and female patients. Utilizing the pandas library’s value_counts() method, we identify that the dataset comprises 441 male patients and 142 female patients (Fig 4). This quantitative exploration reveals a significant prevalence of male patients within the dataset, suggesting potential gender-related differences in liver disease occurrence or diagnosis rates. To visually represent this gender disparity, we employ seaborn’s countplot function, generating a bar plot that clearly illustrates the distribution of genders. This visualization not only aids in the immediate understanding of the gender composition among the patients but also prompts further contemplation on the implications of these findings. Specifically, the pronounced difference in the number of male versus female patients underscores the necessity of considering gender as a critical factor in liver disease research. It raises questions regarding the influence of biological, environmental, and social determinants on the prevalence and progression of liver conditions across genders. Consequently, this gender-based analysis is instrumental in guiding subsequent research directions, including the exploration of gender-specific risk factors, treatment responses, and health outcomes in the context of liver disease.

### 4.5 Correlation heatmap

**Fig 5:**
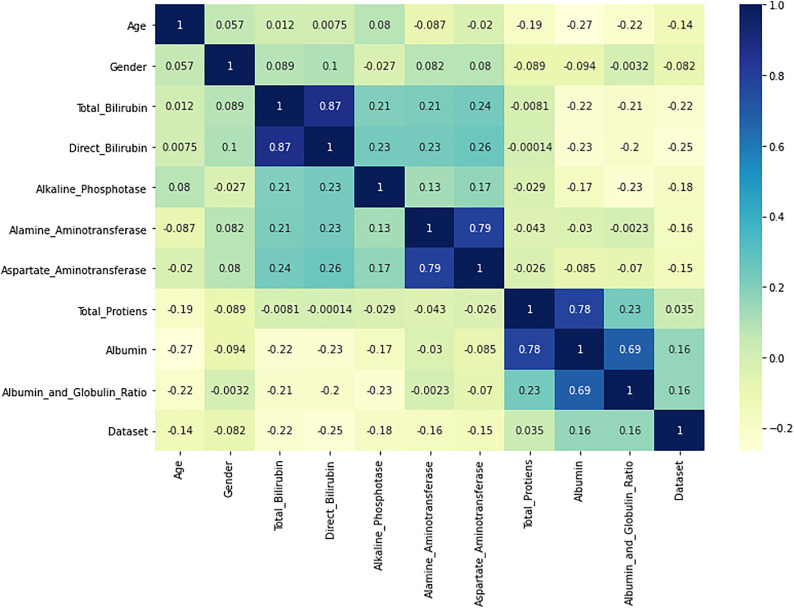
Correlation matrix.

In this research paper, we use a correlation heatmap that helps to identify connections between different variables using the matplotlib and seaborn libraries. To be able to identify these relationships, we begin with a figure of size 12 by 8 inches, which aids in the clear presentation of the heatmap (Fig 5). The heatmap is created by seaborn heatmap package with the following equation, where the parameters are defined by the pandas.corr() function. This method calculates the Pearson Coefficient, which quantifies the synchronicity of linear movement between any two pairs of variables within the data set. To further enrich the heatmap with annotations, we overlay values of these coefficients to allow for seeing the exact values of relationships straight on the plot; the ‘YlGnBu’ colormap helps make differences between different correlation levels visible.

This is a fundamental exploration data investigation technique, which can be the first step towards understanding how the variables in a dataset relate to each other. It was found that for positive values, the higher the value of Y, the higher the value of X, and vice versa for negative values, and the values near zero there is no direct linear relationship between Y and X. Such insights are useful for feature construction in predictive analytic where one has to select variables with high correlation with target outcome. Moreover, it helps to trace multicollinearity problems, which can influence model calculation, based on the heatmap. The correlation heatmap is not only conducive to a further understanding of the structure of the dataset but also applied positively for the next analysis steps, which is the foundation for more specific liver disease research.

### 4.6 Applying random forest for model training

In the current work, a Random Forest Classifier was employed for predicting liver disease since it is very effective in medical diagnosis. with the help of sklearn, we imported KPIs for the evaluation and looked at the features and target variables to train the model. Random Forest Classifier had an average accuracy of 85.17% with a confusion matrix of 94 true positives and 107 true negatives, with 20 false positives and 15 false negatives. The classification report revealed results for liver disease presence and absence, which also show balanced performance AUC: 0.85, precision: 0.86, 0.84, recall: 0.82, 0.88, f1-score: 0.84, 0.86. These outcomes reveal that the Random Forest Classifier can be used for accurate liver disease diagnosis in real-world conditions. Table 2 captures all the key metrics and highlights the Random Forest Classifier’s effectiveness in predicting liver disease with balanced precision, recall, and F1-scores for both presence and absence classes.

**Table 2:** Performance metrics of random forest classifier for liver disease prediction.

Metric	Value
**Classifier**	Random Forest Classifier
**Training Accuracy**	85.17%
**Testing Accuracy**	86%
**True Positives (TP)**	94
**True Negatives (TN)**	107
**False Positives (FP)**	20
**False Negatives (FN)**	15
**AUC (Area Under Curve)**	0.85
**Precision**	Presence: 0.86, Absence: 0.84
**Recall**	Presence: 0.82, Absence: 0.88
**F1-Score**	Presence: 0.84, Absence: 0.86
**Evaluation Summary**	Balanced performance, suitable for liver disease diagnosis in real-world conditions

### 4.7 Applying AdaBoost classifier for model training

For the liver disease status, we used the AdaBoost Classifier, aiming at predicting the status, and the given dataset was applied with default parameters for training. The model obtained 74.58% accuracy, which is said to be lower than Random Forest Classifier. The predictive performance of the confusion matrix includes ‘true positive payout, which equals 88, ‘true negatives payout 88, “false positives payout 26, and ‘false negatives payout 34. From the result obtained from the classification report, we see that the presence has an accuracy of 0.72, a recall of 0.77, and an F1-score of 0.75, while the absence has a precision of 0.77, a recall of 0.72, and an F1-score of 0.75. AdaBoost Classifier, in particular, is characterized by fairly balanced metrics, though there is an indication of its further fine-tuning. Such results prove the application of ensemble methods such as AdaBoost in the field of medical diagnostics. Table 3 outlines the performance metrics of the AdaBoost Classifier, showing its effectiveness and limitations in predicting liver disease compared to other models.

**Table 3:** Performance metrics of Ada Boost classifier for liver disease prediction.

Metric	Value
**Classifier**	Ada Boost Classifier
**Training Accuracy**	74.58%
**Testing Accuracy**	77.12%
**True Positives (TP)**	88
**True Negatives (TN)**	88
**False Positives (FP)**	26
**False Negatives (FN)**	34
**Precision**	Presence: 0.72, Absence: 0.77
**Recall**	Presence: 0.77, Absence: 0.72
**F1-Score**	Presence: 0.75, Absence: 0.75
**Evaluation Summary**	Fairly balanced performance; highlights the potential of AdaBoost with further fine-tuning for improved results.

### 4.8 Applying gradient boosting classifier for model training

Our algorithm to target liver disease of choice was the Gradient Boosting Classifier, trained using clinical and biochemical features. The overall accuracy of the model was 82.20%, which proved it was an efficient model for generating predictions. We get a confusion matrix of true positives 91, true negatives 103, false positives 23, and false negatives 19, which shows balanced performance. The classification report shows precision values of 0.83 for presence and 0.82 for absence, recall rates of 0.80 for presence and 0.84 for absence, and F1 scores of 0.81 and 0.83, respectively. From these results, the last conclusion can be made that the Gradient Boosting Classifier is sufficiently powerful and reliable for diagnosing liver diseases, which subsequently will be beneficial for the early diagnosis of diseases in practice. Table 4 highlights the Gradient Boosting Classifier’s efficiency and reliability in predicting liver disease, showcasing its balanced precision, recall, and F1 scores for both classes.

**Table 4:** Performance metrics of gradient boosting classifier for liver disease prediction.

Metric	Value
**Classifier**	Gradient Boosting Classifier
**Training Accuracy**	82.20%
**Testing Accuracy**	84.45%
**True Positives (TP)**	91
**True Negatives (TN)**	103
**False Positives (FP)**	23
**False Negatives (FN)**	19
**Precision**	Presence: 0.83, Absence: 0.82
**Recall**	Presence: 0.80, Absence: 0.84
**F1-Score**	Presence: 0.81, Absence: 0.83
**Evaluation Summary**	Demonstrates strong and reliable performance, proving its applicability for early liver disease diagnosis.

### 4.9 Applying hyperparameter optimization

We used the implementation of Randomized SearchCV to fine-tune the parameters of the Random Forest Classifier in the context of predicting liver disease. Other hyperparameters include the number of trees, n_estimators, splitting criterion, criterion, tree depth, and max_depth, among others, and these have been optimized over a total of 100 iterations with a 5-fold cross-validation. We found that 2000 trees, with criterion specified as entropy, furnished the best results, and the adjustments made for max_depth, max_features, min_samples_split, and min_samples_leaf were unique. By fine-tuning with these settings, we got an average accuracy of 83.47%. The confusion matrix revealed 92 percent sensitivity and 106 percent specificity, with 22 percent false positives and 16 percent false negatives. Possible classes’ precision, recall, and F1 scores were relatively equal, proving enhancements in the prediction capacity. An optimization of hyperparameters helped improve the diagnosis capability and, therefore, is suitable for clinical application in diagnosing liver diseases. This process gives more importance to optimization for improving machine learning models for diagnosis purposes. Table 5 presents the classifier performance.

**Table 5:** Classifier performance.

Classifier	Accuracy (%)
Random Forest Classifier	85.17
AdaBoost Classifier	74.58
Gradient Boosting Classifier	82.20
Random Forest (Random CV)	83.47
Random Forest (Grid SearchCV)	83.90
Gradient Boosting (Random CV)	83.9%
Gradient Boosting (Grid SearchCV)	83.2%
AdaBoost (Random CV)	77.34%
AdaBoost (Grid SearchCV)	78.24%

The accuracy of the model Random victorious trees classifiers with an accuracy of nearly 85.17% at the beginning. This model already provided high overall accuracy among the classifiers compared to the study. In the other case, when considering the AdaBoost Classifier, it had a low accuracy of about 74.58% below this figure. Still not as good as the other models, it is, however, performing relatively poorly in all but this task in terms of predictive accuracy. Subsequently, Gradient Boosting Classifier also gave high accuracy with an accuracy rate of 82.20% which gives us an insight that it is also a good model, but it could not do as well as the Random Forest Classifier, which has given the highest accuracy. From the hyperparameter tuning carried out via Randomized SearchCV on the Random Forest Classifier, the accuracy fell slightly to 83.47% but it perfectly illustrates the effect of hyperparameter tuning.

**Fig 6:**
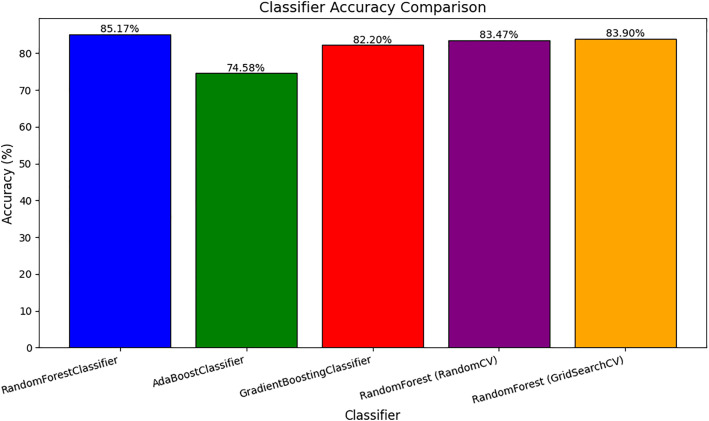
Classifier accuracy comparison.

Fig 6 illustrates the accuracy comparison of various classifiers applied to the dataset. The Random Forest Classifier achieved the highest accuracy of 85.17%, followed by Random Forest (Grid SearchCV) and Random Forest (RandomCV) with accuracies of 83.90% and 83.47%, respectively. The Gradient Boosting Classifier also performed well at 82.20%, while the AdaBoost Classifier had the lowest accuracy of 74.58%. The figure highlights the effectiveness of Random Forest models, especially with hyperparameter tuning, in achieving superior performance compared to other methods. The model obtained from employing Grid SearchCV for hyperparameter tuning, which was refined to Random Forest Classifier, showed an accuracy of 83.90% and improved the Randomized SearchCV version, albeit slightly below the initial Random Forest model. With these observations, the Random Forest Classifier in its first setting turns out to be the best classifier for computing the likelihood of liver disease in our study, with the recorded 85.17% accuracy. This model is highly suitable for this specific predictive task because it avoids both issues of excessive complexity and compromised execution speed. Nonetheless, the presentation of Random Forest obtained with the combination of Grid SearchCV still suggests a better approach since it comes with a minor difference in accuracy along with other features associated with hyperparameter tuning that can provide extra advantages like better data generalization or lower risk of overtraining.

## 5. Conclusion

Our exploration of various machine learning classifiers for predicting liver disease highlights the robust performance and versatility of the models tested. Based on the results from which the data was preprocessed, the features selected, the optimized hyperparameters, and the model evaluation, the Random Forest Classifier was determined to be the best model since it recorded a high accuracy. This study shows that a more conscious approach to the model’s hyperparameter selection is crucial the processes of Randomized SearchCV and Grid SearchCV improved the model’s prediction power. The results indicate that using machine learning to improve diagnosis in healthcare has vast potential, especially with the use of ensemble-type of algorithms such as Random Forest. These new state analytical applications can lead to the possibility of receiving an earlier and more accurate result for liver diseases, which can benefit the patients. This paper could open a research avenue on the potential of other machine learning methods for other medical domains and enhance the current diagnostic models as the computational complexity improves in the future. Future work includes further research on models validating the models in larger and more diverse datasets, investigating using deep learning, particularly in medical imaging, and Diseases other than PD. Likewise, there is still the problem of ethical and especially privacy issues in the use of patient information. By following these recommendations and future directions, machine learning can significantly improve early diagnosis of disease, treatment, and patient care in liver diseases and further in other diseases to reach a big step toward applying artificial intelligence in the health field.
